# Maternal Screen-Related Behaviors, Toddler Screen Use, and Toddler BMI in Mexican American Families: Cross-Sectional Study

**DOI:** 10.2196/76873

**Published:** 2026-02-19

**Authors:** Darcy A Thompson, Laura K Kaizer, Sarah J Schmiege, Natasha J Cabrera, Lauren Clark, Haley Ringwood, Estefania Miramontes Valdes, Andrea Jimenez-Zambrano, Carol Gorman, Marko Babiak, Jeanne M Tschann

**Affiliations:** 1 Department of Pediatrics, School of Medicine, University of Colorado Anschutz Medical Campus, 12631 East 17th Avenue, Aurora, CO, 80045, United States, 1 720-777-2715; 2 Adult & Child Center for Outcomes Research and Delivery Science, School of Medicine, University of Colorado Anschutz Medical Campus, Aurora, CO, United States; 3 Department of Biostatistics and Informatics, Colorado School of Public Health, University of Colorado Anschutz Medical Campus, Aurora, CO, United States; 4 College of Education, University of Maryland, College Park, College Park, MD, United States; 5 School of Nursing, University of California, Los Angeles, Los Angeles, CA, United States; 6 Denver Health and Hospital Authority, Denver, CO, United States; 7 Department of Family Medicine, School of Medicine, University of Colorado Anschutz Medical Campus, Aurora, CO, United States; 8 Department of Psychiatry and Behavioral Sciences, University of California, San Francisco, San Francisco, CA, United States

**Keywords:** restriction, digital media, behavior management, technology, early childhood

## Abstract

**Background:**

Parents, as the most proximal influence on young children, play an important role in shaping toddler behaviors. Yet, evidence on how parents shape toddler screen use is limited. Little is also known about the relationship between toddler screen use and BMI. Given existing disparities in screen use and early childhood obesity, a focus on Mexican American families with toddlers is warranted.

**Objective:**

This study aimed to evaluate the independent contributions of both maternal screen use and screen-related parenting practices with toddler screen use duration, for both TV viewing and mobile device use, and examine the relationship between toddler screen use duration and BMI.

**Methods:**

This cross-sectional study enrolled 384 Mexican American mother-toddler dyads recruited from safety net clinics. Enrolled mothers completed 7-day screen use diaries and surveys on screen-related parenting practices, and toddler anthropometrics were obtained. Negative binomial regression models estimated the relationships between screen-related parenting practices and maternal screen use (predictors) with child duration of daily TV use and mobile device use (outcomes). Spearman correlations were calculated to estimate the relationship between toddler screen use duration and age- and sex-specific BMI *z* scores.

**Results:**

Maternal duration of daily TV and mobile device use were associated with toddler duration of daily TV (adjusted rate ratios [aRRs] 1.27‐1.28; all *P*<.001) and mobile device use (aRRs 1.17‐1.18; all *P*<.001), respectively, even after adjusting for maternal screen-related parenting practices. Specific parenting practices, including restriction of TV time (aRR=0.86; *P*=.01), restriction of mobile device time (aRR=0.80; *P*=.02), use of TV (aRR=1.27; *P*=.003) and mobile devices (aRR=1.78; *P*<.001) for child behavior regulation, and coviewing of mobile devices (aRR=1.51; *P*<.001), were associated with toddler duration of daily screen use, adjusted for maternal duration of daily screen use. Neither toddler duration of daily TV viewing nor daily mobile device use was correlated with toddler BMI *z* scores*.*

**Conclusions:**

Both the duration of maternal screen use and screen-related parenting practices, for both TV and mobile devices, should be considered when promoting healthy screen use in toddlers in Mexican American families. Interventionists should consider the family ecology when designing interventions promoting healthy screen use in early childhood.

## Introduction

While the American Academy of Pediatrics (AAP) and numerous other organizations have clearly outlined recommended parameters for screen use in children aged younger than 3 years [1-3], effective interventions promoting healthy screen use, such as limited duration of use and no use before bedtime, are lacking for this age group [1,4,5]. This is in part due to the limited evidence informing the design of such interventions [1,4,6,7]. Our understanding of the contributors to screen use in toddlers is lacking, even though screen use often starts in the first year of life, with early screen use behaviors often persisting throughout childhood [8,9]. Given the impact of screen use on the well-being of young children, both positive and negative, there is a need to identify modifiable factors contributing to screen use in the first few years of life [10-12].

Numerous experts have called for a focus on the family ecology around screen media use, particularly in early childhood [10,13-16]. Family systems theory [17], which recognizes the interconnectedness and interdependence of family members and the emergence of behavior patterns within a family system, further suggests the need to evaluate the relationship of screen-related parental behaviors with child screen use. Parents, as the most proximal influence on young children, play an important role in shaping their toddlers’ screen use [18]. Evidence suggests that certain screen-related parenting practices (eg, restriction of use or coviewing) are associated with child screen use duration [16,19-22]. In our own previous work with Mexican American families with preschoolers, maternal restriction of duration of TV viewing was associated with fewer minutes of preschooler daily TV viewing [23]. A small study in Canada of mainly White and middle-class families with 1.5- to 5-year-olds as well as a larger study in Israel of parents of toddlers (aged 1.5‐3 y) reported that greater maternal use of screens to manage child behaviors was associated with increased screen use duration [21,24]. While screen-related parenting practices are important, parents’ own screen use, specifically the duration of their screen use, is also associated with child screen use in children younger than 5 years [25-28]. Given the interconnectedness of members within families, children may learn to use screen devices by observing parental behaviors. Moreover, in households with limited space, children may end up watching TV as a natural result of their parents deciding to watch TV themselves. Altogether, these studies suggest multiple ways in which parents shape child screen use behaviors.

Although the evidence on the influence of parent screen-related behaviors on toddler screen use is growing, important limitations of existing research need to be addressed. To start, most of these studies are limited by imprecise measurement of parent and child screen use, using global parental reports to measure screen use, which are not highly correlated with actual screen use [29-31]. Additionally, most studies use 1- or 2-item measures of screen-related parenting, which tend to have poor reliability, and for which validation studies are lacking [21,24,32]. Moreover, only 1 study, to our knowledge, has evaluated the independent associations of parental screen use and screen-related parenting practices with the duration of toddler screen use, finding that both independently predicted child screen time [33]. Parental restriction of screen time was associated with reduced toddler screen time, and parent screen time was associated with increased toddler screen time [33]. Work is needed to clarify whether interventions promoting healthy screen use in toddlers should focus on parents’ own screen use in addition to the typical focus on screen-related parenting practices. Addressing these measurement and design issues is important to ensure that future interventions can be designed to focus on modifying the most important influences on toddler screen use.

An additional limitation of existing research is that most studies elucidating the role of parents in shaping screen use in toddlers measure all types of screen use as a single category, even though toddlers interact with multiple different types of screens, including television, smartphones, and tablets [34]. As a result, investigators have been unable to consider whether relationships between parental screen-related behaviors and child screen use vary by screen device type. In our previous work, we found that parental perceptions of the risks and benefits of screen devices can depend on the type of device, which may impact how they parent regarding that device [35]. For example, some parents reported that they needed to pay closer attention to the content their child views on a mobile device compared to TV, and they more commonly used mobile devices for child behavior management than TV [35]. Moving forward, measurement of screen-related parenting should consider variation in parenting across certain screen types to ensure a more nuanced understanding of parenting.

While evidence is growing regarding the impact of screen use on toddler well-being, to date, little is known about whether toddler screen use is associated with BMI [36]. Possible mechanisms for this relationship include increased sedentary time secondary to limited movement while viewing; altered dietary intake, either because screen use while eating distracts one from recognizing satiety cues or screen content (eg, food and beverage commercials) influences food preferences; and reduced sleep duration, potentially resulting from stimulation caused by screen use before bed [36,37]. In children aged 3 years and older, more screen time is associated with obesity [37]. Yet, for children aged younger than 3 years, only a handful of studies exist. Overall, findings are mixed for the studies evaluating the association of toddler screen use with BMI during the toddler years [38-42]. Longitudinal studies, however, suggest that toddler screen use duration does predict BMI years later [43-46]. Yet, again, most of these studies rely on imprecise measurement of screen use (usually a few survey questions). While obesity is multifactorial in origin, understanding the relationship between toddler screen use and toddler weight is needed to inform interventions aiming to prevent early childhood obesity [47]. 

Focusing future work on Latino children is important because, as a group, they are disproportionately affected by obesity starting in very early life [48-51]. Evidence suggests that approximately 15% of children younger than 2 years and approximately 16% of Latino children aged 2 to 5 years meet the criteria for obesity [48,52]. Unhealthy screen use (eg, higher duration of use) is also more common in low-income Latino children than higher income and non-Latino White children [53,54]. Recognizing the cultural heterogeneity of Latino populations [55,56], our work focuses on the largest subgroup of Latinos, Mexican Americans [48-51,57,58]. Approximately 16% of children in the United States are of Mexican heritage. Results of this work may therefore be generalizable to a large portion of children in the United States [59,60].

In this study, we aimed to advance our understanding of family-level contributors (ie, maternal screen use and screen-related parenting practices) to toddler screen use in Mexican American families by addressing the above-outlined limitations in the existing literature. We addressed limitations in the measurement of screen use in previous studies by using diaries to measure screen use duration. Screen use diaries are known to be highly correlated with actual screen use and allow measurement of screen use across device types [30,61]. We also used multi-item measures of screen-related parenting practices that we developed through a systematic process, applying a mixed methods approach and allowing for measurement of device-specific parenting [62]. We expected that maternal screen use and screen-related parenting practices would be independently associated with toddler screen use duration, for both TV viewing and mobile device use. Additionally, we examined the association between toddler screen use duration and BMI to expand existing evidence on this topic in toddlers. Findings will help to inform the design of interventions promoting healthy screen use in Mexican American families with toddlers.

## Methods

### Ethical Considerations

This study was part of a larger cross-sectional study aiming to evaluate contributors to and outcomes of early childhood screen use in Mexican American communities. The Colorado Multiple Institutional Review Board approved this study (18‐1662; initial approval 2018). All participants gave informed consent, documented with an electronic or written signature, before enrolling in the study. Data are stored securely to ensure confidentiality. Participants were compensated with gift cards: US $95 (mother) and US $45 (toddler).

### Recruitment

A convenience sample of 384 families was recruited from a safety-net health care system in Denver, Colorado (November 2020 to December 2023). Spanish- and English-speaking parents of toddlers identified as Latino or Spanish-speaking in the electronic medical record and living in the greater Denver, Colorado, metropolitan area were sent an introductory letter about the study. Letters were followed by phone calls with the child’s mother (inclusive of caregivers in maternal roles) to assess interest and eligibility. Mothers were eligible if they were at least 18 years old, identified as being of Mexican heritage, had screen devices at home, and denied having a condition that limited the way they used screen devices. Additionally, their 15‐ to 26-month-old child had to be ambulatory, live with them most of the time, and not have a health or developmental condition that impacted their child’s sleep, physical activity, diet, or growth. Adult participants in this study are labeled “mothers” since 383 (99%) of 384 were the focal child’s birth mother. Of the 773 eligible mothers, 384 (49.7%) completed data collection in this study.

### Procedures

Informed consent was conducted by phone by trained bilingual and bicultural team members. Consent documents were sent electronically via text or email based on participant preference, with participant signatures being captured electronically, or in some instances, in person. Following informed consent, mothers completed a 1- to 1.5-hour phone interview in which trained bilingual and bicultural study staff administered surveys. In the days following the first survey, staff dropped off and trained mothers on the completion of the 7-day screen use diary and conducted anthropometric measurements on toddlers. Following the pick-up of the diary, staff administered another 1‐ to 1.5-hour phone interview to complete the final survey items. Survey and anthropometric data were collected and managed using REDCap (Research Electronic Data Capture) tools hosted at the University of Colorado [63]. All communication was conducted in the preferred language of the participant.

Survey items were evaluated and adapted as needed to ensure relevance and clarity for the enrolled sample. Survey items were translated by a bilingual/bicultural team member into Spanish or English as needed. We compared the 2 language versions side by side as a group consisting of bilingual investigators and research staff. We evaluated translations for conceptual equivalence and contextual and cultural relevance, applying a decentering process in which alterations were made to either language version to obtain conceptual equivalence [64,65]. We then pretested items using cognitive interviews in both Spanish and English to ensure easy comprehension as well as shared conceptual meaning across participants and the investigative team [66]. Details of this process are described elsewhere [62,67]. Finally, following in-depth training of the research team, we piloted study procedures with 7 mother-toddler dyads, prior to starting data collection.

### Measures

#### Outcomes

##### Toddler Duration of Daily TV and Mobile Device Use

Mothers completed a written 7-day daily screen use diary tracking their toddler’s TV and mobile device use (smartphones and tablets) in 15-minute increments from 5 AM to midnight, similar to other studies [61,68]. Daily TV and mobile device use amounts in minutes were calculated and then averaged over the number of diary days for those submitting at least 5 days of diary data. Screen use diaries are highly correlated (*r*=0.84) with the actual amount viewed [30,61], overcoming the limitations of most screen use research that was based on parent global estimates of screen use. Because screen use can change day to day, mothers were asked to complete a full 7-day diary.

##### Toddler Age- and Sex-Specific BMI *Z* Score

Trained staff measured length in children younger than 24 months or height for those 24 months or older to the nearest 0.1 cm and weight (light clothing and no shoes) to the nearest 0.01 kg, according to recommendations, using portable digital scales (Seca 354) and measuring boards (Seca 417) or stadiometers (Seca 217) [69]. When it was not feasible to obtain a length in those younger than 24 months or a height in those 24 months or older, we obtained the other measurement, that is, a height or length, respectively, and added or subtracted the standard 0.7 cm [69]. Age- and sex-specific BMI *z* scores were calculated based on the World Health Organization growth charts [70]. BMI is a typical measure of weight status in research studies on this age group [71-75]. Toddler BMI is associated with both adiposity and risk of future obesity [71-75]. Due to COVID-19 pandemic–related restrictions, some toddlers’ anthropometrics were not collected or were collected more than 30 days following completion of the surveys and diaries. For this study, toddlers whose anthropometric data were collected more than 30 days from the date of the first survey were excluded from analyses.

### Predictors: Maternal Screen-Related Behaviors

#### Maternal Duration of Daily TV and Mobile Device Use

Mothers completed a written 7-day daily screen use diary tracking their own TV and mobile device use (smartphones and tablets) in 15-minute increments from 5 AM to midnight. Daily TV and mobile device use amounts were calculated and then averaged over the number of diary days for those submitting at least 5 days of diary data. Mothers completed their own screen use diary the same week they completed their child’s diary to capture contemporaneous patterns of mother and child screen use. To minimize the possibility of inflated associations, the diaries were designed with separate pages for mother and child and physically bound together in a way that prevented easy side-by-side comparison during completion.

#### Maternal Screen-Related Parenting Practices

Seven domains of screen-related parenting practices were measured; restriction of toddler screen time (TV use: 8 items, *α*=.82; mobile device use: 8 items, *α*=.84), use of screens for behavioral regulation (eg, have their child use a screen to calm down or when misbehaving, TV use: 12 items, *α*=.90; mobile device use: 16 items, *α*=.91), coviewing screens with the child (TV use: 10 items, *α*=.86; mobile device use: 10 items, *α*=.87), and restriction of content viewed on screens, with screens defined as both TV and mobile devices (8 items, *α*=.83). Device-related parenting behaviors were not administered to participants who said the focal child had never used the device type (TV or mobile devices), because for these children, no device-specific parenting behaviors occurred. Item response options ranged from never (0) to always (4) on a 5-point Likert scale, with higher values indicating greater endorsement of the item (ie, greater content restriction, greater coviewing). Methods for the development of these measures are described in detail elsewhere [62]. Briefly, items were developed from findings from 32 semistructured interviews with Mexican American parents of toddlers aged 15 to 26 months [35,76], followed by exploratory and confirmatory factor analyses using a split sample approach.

### Covariates

Mothers reported child age in months, child gender, maternal age, education level, partnership status (ie, married/partnered or not), employment status (yes/no), number of children in the home, and acculturation. Acculturation was measured using the Bidimensional Acculturation Scale for Hispanics: non-Hispanic acculturation scale (12 items; *α*=.97) [77]. Scores range from 1 to 4, with higher scores reflecting higher levels of acculturation. To capture the possible impact of the COVID-19 pandemic on everyday life throughout the period of data collection, we created a binary variable that reflected whether the Denver County Department of Health had any COVID-19 pandemic–related restrictions in place at the time of data collection, coded as no restrictions in place=1 and any restrictions in place=0.

### Analysis

Analyses were completed in SAS Version 9.4 (SAS Institute). From the sample of 384 participants, we removed those mother-child dyads who were missing duration of daily screen use (mothers n=2; child n=8), and those with toddler anthropometrics collected for more than 30 days following survey administration (n=61), resulting in a sample size of 313. In addition, 20% (n=62) of children did not use a mobile device, and thus, parents were not asked the mobile device–specific parenting practices questions. Accordingly, for analyses involving mobile devices, the sample size was 251. Due to the skewness of some variables, Spearman correlations were calculated between covariates, maternal screen-related behaviors, and outcomes to determine bivariate associations among variables. Because the outcome data were counts, negative binomial regression was used to estimate the relationships between maternal screen use and screen-related parenting practices (predictors) with child duration of daily TV use and mobile device use (outcomes). Regression modeling was carried out separately for TV use and mobile screen use. Three different regression models were estimated for each due to the substantial correlations between some parenting practices. All models included maternal screen use duration, with model 1 estimating restriction of time and content, model 2 estimating behavioral regulation, and model 3 estimating coviewing. Maternal restriction of time and content were included in the same model because, although they represent distinct parenting practices, they are complementary strategies widely recommended to promote healthy screen use in young children [1]. Including both practices in the same model enabled us to examine their independent associations with child screen use duration. Covariates associated with either TV or mobile use at *P*<.2 were included in all models. Final results are presented as adjusted rate ratios. Rate ratios greater than 1 indicate increased screen use for every 1 unit increase in the predictor, while rate ratios less than 1 indicate decreased screen use for every 1 unit increase in the predictor. Maternal duration of daily TV and mobile device use were converted to hours for regression modeling to improve the interpretability of the rate ratios.

## Results

Mothers were on average 31 (SD 6.0) years old, and the majority were partnered (264/313, 84.3%; Table 1). Toddlers were on average 21.2 (SD 3.0) months old, and nearly half (153/313, 48.6%) were male participants. The majority of surveys were administered in Spanish (241/313, 77.0%). Toddler average daily TV use was 94.3 (IQR 49.3‐173.6) minutes, and average daily mobile use was 27.9 (IQR 6.4‐66.4) minutes. There was no significant correlation between average daily TV use and average daily mobile use (ρ=0.09; *P*=.15).

**Table 1.** Characteristics of Mexican American mother–toddler dyads recruited from safety-net clinics.

Characteristic	Values
Child characteristics (n=313)	
Age (mo), mean (SD)	21.2 (3.0)
Male sex, n (%)	152 (48.6)
Maternal characteristics (n=313)	
Age (y), mean (SD)	31.3 (6.0)
Education (y), mean (SD)	11.6 (2.5)
Partnered, n (%)	264 (84.3)
Employed, n (%)	130 (41.5)
Acculturation: non-Hispanic, median (IQR)	2.2 (1.7 to 3.2)
Number of children in home, median (IQR)	3.0 (2.0 to 3.0)
No COVID-19–related restrictions in place, n (%)	259 (82.7)
Toddler screen use	
Duration of daily TV (min; n=313), median (IQR)	94.3 (49.3 to 173.6)
Child ever used a mobile device, (n=313), n (%)	251 (80)
Duration of daily mobile device use (min; n=251), median (IQR)	27.9 (6.4 to 66.4)
Screen-related parenting practices	
Television^a^ (n=307), median (IQR)	
Restrict time	2.3 (1.6 to 2.9)
Behavior regulation	0.9 (0.6 to 1.3)
Coview	2.0 (1.4 to 2.5)
Mobile device use^b^ (min; n=251), median (IQR)	
Restrict time	2.5 (1.8 to 3.1)
Behavior regulation	0.7 (0.4 to 1.0)
Coview	1.2 (0.9 to 1.7)
Screen use (n=312), median (IQR)	
Content restriction	3.6 (3.2 to 4.0)
Maternal screen use (n=313), median (IQR)	
Duration of daily TV (min)	75.0 (32.1 to 145.7)
Duration of daily mobile device use (min)	145.7 (70.7 to 246.4)
Toddler anthropometrics (n=274), median (IQR)	
BMI *z* score^c^	0.7 (–0.1 to 1.5)

a Reported only for children who ever viewed television (n=307).

b Reported only for children who have ever used a mobile device (n=251).

c Not reported in some due to the impact of COVID-19 on ability to collect or timing of collection (>30 d from survey administration).

Spearman correlations between maternal screen-related behaviors (screen use duration and parenting practices), child screen use duration, child BMI *z* scores, and covariates included in the final regression models are presented in Table 2 for TV viewing and Table 3 for mobile device use. Neither toddler duration of daily TV viewing nor daily mobile device use was correlated with toddler BMI *z* scores. Similarly, average total duration of screen use, inclusive of TV and mobile device use, was not correlated with BMI *z* scores (ρ=0.06; *P*=.30).

**Table 2.** Correlation matrix for screen-related parenting practices, maternal TV use, child TV use, and child BMI *z* scores (n=313).

	Screen-related parenting practices				Mother	Child		Covariates		
	Television use			Screens	Duration of daily TV (min)	Duration of daily TV (min)	BMI *z* score	Child age (mo)	Child gender	No COVID-19–related restrictions
	Restrict time	Coview	Behavioral regulation	Restrict content						
Restrict time										
* r*	1.00									
*P* value	<.001									
Coview										
* r*	0.21	1.00								
*P* value	<.001	<.001								
Behavioral regulation										
* r*	–0.28	0.26	1.00							
*P* value	<.001	<.001	<.001							
Restrict content										
* r*	0.39	0.18	–0.20	1.00						
*P* value	<.001	.002	<.001	<.001						
Mother duration of daily TV (min)										
* r*	–0.11	0.18	0.15	0.02	1.00					
*P* value	.05	.002	.009	.73	<.001					
Child duration of daily TV (min)										
* r*	–0.17	0.12	0.28	0	0.60	1.00				
*P* value	.003	.04	<.001	.99	<.001	<.001				
Child BMI *z* score										
* r*	–0.08	0.02	0.11	–0.07	0	0.04	1.00			
*P* value	.21	.77	.08	.25	.97	.47	<.001			
Child age (mo)										
* r*	–0.15	–0.04	0.05	–0.18	–0.01	–0.03	0.03	1.00		
*P* value	.007	.50	.38	.002	.88	.54	.63	<.001		
Child gender										
* r*	–0.08	–0.01	0	0.09	0.04	–0.06	0.03	0.07	1.00	
*P* value	.15	.86	.93	.12	.53	.33	.58	.19	<.001	
No COVID-19–related restrictions										
* r*	–0.02	0	0.09	–0.7	0.01	0.11	–0.02	0.09	0.05	1.00
*P* value	.67	>.99	.10	.22	.85	.06	.70	.10	.41	<.001

**Table 3.** Correlation matrix for screen-related parenting practices, maternal mobile device use, child mobile device use, and BMI *z* scores (n=251).

	Screen-related parenting practices				Mother	Child		Covariates		
	Mobile Device Use			Screens	Duration of daily mobile use (min)	Duration of daily mobile use (min)	BMI *z* score	Child age (mo)	Child gender	No COVID-19–related restrictions
	Restrict time	Coview	Behavioral regulation	Restrict Content						
Restrict time										
* r*	1.00									
*P* value	<.001									
Coview										
* r*	–0.01	1.00								
*P* value	.93	<.001								
Behavioral regulation										
* r*	–0.40	0.42	1.00							
*P* value	<.001	<.001	<.001							
Restrict content										
* r*	0.42	0	–0.26	1.00						
*P* value	<.001	.97	<.001	<.001						
Mother duration of daily mobile use (min)										
* r*	–0.14	0.07	0.16	–0.10	1.00					
*P* value	.02	.26	.01	.12	<.001					
Child duration of daily mobile use (min)										
* r*	–0.28	0.29	0.38	–0.13	0.31	1.00				
*P* value	<.001	<.001	<.001	.04	<.001	<.001				
Child BMI *z* score										
* r*	–0.1	0.05	0.22	–0.06	0.04	0.06	1.00			
*P* value	.13	.43	.001	.37	.54	.36	<.001			
Child age (mo)										
* r*	–0.15	0.07	0.22	–0.15	0.01	0.20	–0.02	1.00		
*P* value	.02	.27	<.001	.02	.90	.001	.75	<.001		
Child gender										
* r*	–0.03	–0.01	0.06	0.11	0.04	0.08	0.03	0.09	1.00	
*P* value	.60	.93	.34	.09	.53	.20	.70	.17	<.001	
No COVID-19–related restrictions										
* r*	–0.02	0.02	0.11	–0.08	0.10	0.09	–0.02	0.11	0.05	1.00
*P* value	.76	.79	.07	.19	.10	.15	.82	.09	.41	<.001

Results of the negative binomial regression models estimating the relationships between maternal screen-related behaviors with toddler duration of daily TV use and daily mobile device use are presented in Table 4. In the regression analyses specific to television use, all models included maternal duration of daily TV (h). Model 1 included the restriction of toddler duration of TV use (restrict time) and restriction of content viewed (restrict content). Model 2 included the parenting practice of using TV for toddler behavior regulation (behavioral regulation), and model 3 included coviewing of TV (coview). Similar models were used in the analysis focused on mobile device use, with variables specific to mobile device use. Findings were similar across models for TV use and mobile device use.

Across all models shown in Table 4, maternal duration of daily screen use variables (TV and mobile device use) were associated with toddler duration of daily screen use for both TV and mobile device use, respectively, even after adjusting for maternal restriction of toddler screen time and restriction of content viewed, use of screen devices for behavioral regulation, and coviewing (Table 4, all *P*<.001).

Parenting practices were also associated with toddler duration of daily screen use, independent of maternal duration of daily screen use. Increased restriction of toddler screen time, for both TV use and mobile device use, was associated with decreased toddler duration of daily screen use (television use—model 1: restrict time *P*=.01 and mobile use—model 1: restrict time *P*=.02 in Table 4), adjusted for both maternal duration of daily screen use and maternal restriction of content viewed on TV or mobile devices. However, restriction of toddler content viewed on TV or mobile devices was not associated with child duration of daily screen use, when adjusted for both maternal duration of daily screen use and maternal restriction of toddler screen time (Table 4; television use—model 1: restrict content *P*=.28 and mobile use—model 1: restrict content *P*=.60). Increased maternal use of screen devices for child behavior regulation was associated with increased toddler duration of daily screen use for both TV use and mobile device use, independent of maternal duration of daily screen use (television use—model 2: behavior regulation *P*<.001 and mobile use—model 2: behavior regulation *P*<.001 in Table 4). Finally, a mother’s practice of coviewing TV with their toddler was not associated with toddler duration of daily TV viewing (television use—model 3: coview *P*=.41), whereas mothers’ greater coviewing of mobile devices with their toddler was associated with increased toddler duration of daily mobile use (mobile use—model 3: coview *P*<.001). Both models were adjusted for maternal duration of daily screen use.

**Table 4.** Negative binomial regression models evaluating relationships between maternal screen-related behaviors and child duration of daily TV use and mobile use (min).

Negative binomial regression models	Adjusted rate ratio (95% CI)	*P* value
Television use		
Model 1		
Maternal duration of daily TV (h)	1.27 (1.21‐1.33)	<.001
PP^a^: restrict time	0.86 (0.77‐0.96)	.01
PP: restrict content	1.08 (0.94‐1.26)	.28
Model 2		
Maternal duration of daily TV (h)	1.27 (1.21‐1.33)	<.001
PP: behavior regulation	1.27 (1.08‐1.49)	.003
Model 3		
Maternal duration of daily TV (h)	1.28 (1.22‐1.35)	<.001
PP: coview	0.95 (0.84‐1.07)	.41
Mobile use		
Model 1		
Maternal duration of daily mobile device use (h)	1.17 (1.08‐1.27)	<.001
PP: restrict time	0.80 (0.66‐0.97)	.02
PP: restrict content	0.93 (0.69‐1.24)	.60
Model 2		
Maternal duration of daily mobile device use (h)	1.17 (1.08‐1.27)	<.001
PP: behavior regulation	1.78 (1.27‐2.49)	<.001
Model 3		
Maternal duration of daily mobile device use (h)	1.18 (1.09‐1.29)	<.001
PP: coview	1.51 (1.18‐1.94)	<.001

a PP: parenting practice.

## Discussion

This cross-sectional study evaluated associations between maternal screen-related behaviors and toddler screen use duration across screen types in Mexican American families recruited from safety-net clinics. Both the duration of maternal screen use and screen-related parenting practices were independently associated with the duration of toddler screen use, suggesting that interventionists should consider both domains of parent behavior when promoting healthy screen use in toddlers. Addressing the lack of evidence on the impact of screen use on toddler well-being, we also evaluated the relationship between toddler screen use duration and toddler BMI but found no relationship between toddler duration of TV viewing or mobile use with BMI. The methods of this study addressed many of the limitations of previous studies. Specifically, we used 7-day diaries to measure parent and toddler screen use duration and used multi-item measures of screen-related parenting practices developed through rigorous methods to reliably measure multiple dimensions of screen-related parenting practices. Given that early screen use behaviors often persist throughout childhood [8,9], interventions that support families, including parents and toddlers, in the healthy use of screen devices are needed.

This study’s finding that both maternal screen use and screen-related parenting practices are related to toddler screen use duration extends previous evidence on this topic. Only 1 other study evaluated both parental screen use and screen-related parenting practices in the same model, assessing the independent effect of each on toddler screen use duration [33]. That study focused on parental restriction of toddler duration of screen use and parental limits on electronics in the bedroom in a socioeconomically diverse sample of families with 18-month-olds in Canada. They found that increased parental restriction of toddler duration of screen use and lower parental duration of screen use were associated with lower durations of toddler screen use [33]. However, their findings are limited due to the use of global estimates of parent and child screen use, inclusive of TV, computers, and video games [33]. Additionally, they did not examine differences by screen device type [33]. Numerous other studies have evaluated either parental screen use [26,33,78-80] or parenting practices in relation to toddler screen use duration, reporting findings similar to ours [16,19-22]. Our study extends the evidence on this topic by evaluating both parental practices and parental screen use together, using enhanced measures of screen use, and evaluating parenting practices specific to screen device type. Altogether, the findings underscore the need for interventions to consider ways to address both parental screen use and screen-related parenting practices in mothers in order to support healthy toddler screen use.

A strength of this study is the focus on Mexican Americans, a cultural group experiencing disparities in unhealthy screen use [53,54], as well as childhood obesity [48-51]. We used methods in this study to ensure rigorous and culturally- and contextually-relevant measurement supporting the validity of the findings [35,62]. While our results may apply to other cultural groups, the evidence provided here could inform the development of interventions aiming to promote healthy screen use in toddlers in Mexican American communities, especially those that are under-resourced. To further enhance the design of such interventions, additional research evaluating how the broader context within which families live and how the related stressors they experience might impact screen-related parenting.

An additional strength of this study is the separate consideration, in both our measurement of screen use and our analytic approach, of TV viewing and mobile device use, thus providing a nuanced evaluation of the relationships between parental screen-related behaviors and toddler screen use. The results suggest that maternal role modeling of both TV viewing and mobile device use is associated with duration of toddler TV viewing and mobile device use, respectively, independent of a variety of device-specific parenting practices. As for screen-related parenting practices, findings were similar for both TV and mobile devices for both time restriction and behavioral regulation with devices. Interestingly, coviewing of mobile devices was associated with increased mobile device use, whereas coviewing of TV was not associated with toddler duration of TV viewing. Prior qualitative work in this population suggests that this could be due to high levels of parental concern for inappropriate content exposure on mobile devices, possibly leading to more coviewing of mobile devices [35]. Moreover, it is possible that when parents are using mobile devices, their toddlers are with them more often than when watching TV. Future research should continue to consider differences in screen-related parental behaviors by type of screen device in use.

The AAP’s recommendations regarding certain parental screen-related behaviors are supported by our findings. The AAP recommends limiting toddlers’ duration of use as well as limiting parental use of screens for behavioral regulation, both of which were associated in our study with decreased screen use duration for both TV and mobile devices. The AAP also recommends coviewing content with children. While our findings support a link between coviewing and child duration of use for mobile devices, but not for TV, there are numerous reasons why parents should coview both TV and mobile device content with their child [1]. Coviewing of content helps parents to ensure that their child is exposed to appropriate content in addition to offering the possibility of scaffolding their child’s learning [1]. The AAP also recommends the use of a “Family Media Plan,” which includes parents setting guidelines for their own use [1]. Our finding that the duration of maternal media use is positively associated with child duration of use supports the need for this plan. That said, counseling parents on their parenting practices across a variety of domains is common in pediatric offices, whereas addressing the entire family’s behaviors is more challenging [81]. Moreover, whether families are receptive to such counseling from their child’s provider is unknown. Research is needed to understand the best approaches for promoting healthy screen use within families, especially given the instrumental support screen devices offer parents in managing child behavior [82,83]. Parental use of screens for behavioral regulation of their toddler is common, offering what is perceived by many parents as an effective way to get things done while with their child [35]. Clearly, intervention work must consider the context of families with young children, parent and child needs, as well as the timing of such intervention.

Similar to the handful of other studies in this area, we did not find a relationship between toddler duration of screen use and BMI [41,42]. Our focus on a specific population and our use of diaries to measure screen use extend the current literature in this area. Proposed mechanisms of the relationship between screen use and a child’s BMI may not be as applicable in the first few years of life. For example, toddlers (1- and 2-year-olds) may not be as sedentary while using screen devices compared to older children. Because early screen use is associated with higher BMIs in the preschool years (ages 3‐5 y) and beyond, intervening on screen use during the first few years of life is still warranted [36].

Limitations of this study warrant mention. To start, this work only focuses on maternal parenting practices despite research highlighting the important role of fathers in managing child screen use [24,35,84]. Second, the cross-sectional design of this study does not provide information on the direction of the relationships we evaluated. Longitudinal data collection would allow for an understanding of the factors contributing to the development of toddler screen use over time. Third, data collection occurred during different phases of COVID-19 restrictions. Other studies captured changes in multiple behaviors, including screen use, during the early stages of the pandemic [85-87]. Accordingly, we adjusted for COVID-19–related restrictions in our models to control for the possibility that COVID-19 affected these relationships. Finally, while the use of diaries for the collection of screen use is an advance over the measures used in current evidence, enhanced measurement is needed. Moreover, while we intentionally asked mothers to complete their own screen use diary during the same week they completed their child’s diary to capture the contemporaneous relationship between mother and child screen use, this approach may have inflated the observed associations. As mentioned previously, we designed the diaries in such a way as to minimize this possibility. Of note, other studies using alternative measures of screen use have noted strong correlations between mother and child screen use, suggesting our finding is not solely an artifact of our measurement approach [25-28]. Recognizing the limitations of self-report measures, investigators are working to develop objective measures of screen use that are feasible and acceptable across diverse populations. One such example is the Family Level Assessment of Screen Use in the Home-Television system, which uses face detection and gaze estimation to measure TV viewing in the home [88].

In summary, both the duration of a mother’s own screen use and screen-related parenting practices should be considered when promoting healthy screen use in toddlers in Mexican American families. This holds true for both TV viewing and mobile device use. While these findings support the AAP’s recommendations for families, additional research is needed to understand the role of fathers and to identify effective ways for providers to support families in following these guidelines. Furthermore, the development of effective evidence-based interventions that are culturally and contextually relevant is needed.

## Abbreviations

APP: American Academy of Pediatrics
aRR: adjusted rate ratio
REDCap: Research Electronic Data Capture
